# Assessment of Tidal Range Changes in the North Sea From 1958 to 2014

**DOI:** 10.1029/2020JC016456

**Published:** 2021-01-18

**Authors:** Leon Jänicke, Andra Ebener, Sönke Dangendorf, Arne Arns, Michael Schindelegger, Sebastian Niehüser, Ivan D. Haigh, Philip Woodworth, Jürgen Jensen

**Affiliations:** ^1^ Research Institute for Water and Environment University of Siegen Siegen Germany; ^2^ Centre for Coastal Physical Oceanography Old Dominion University Norfolk USA; ^3^ Faculty of Agricultural and Environmental Sciences University of Rostock Rostock Germany; ^4^ Institute of Geodesy and Geoinformation (IGG) University of Bonn Bonn Germany; ^5^ School of Ocean and Earth Science University of Southampton Southampton UK; ^6^ National Oceanography Centre Liverpool UK

**Keywords:** amphidromes, empirical orthogonal function (EOF), Kriging, North Sea, stratification, tidal range

## Abstract

We document an exceptional large‐spatial scale case of changes in tidal range in the North Sea, featuring pronounced trends between −2.3 mm/yr at tide gauges in the United Kingdom and up to 7 mm/yr in the German Bight between 1958 and 2014. These changes are spatially heterogeneous and driven by a superposition of local and large‐scale processes within the basin. We use principal component analysis to separate large‐scale signals appearing coherently over multiple stations from rather localized changes. We identify two leading principal components (PCs) that explain about 69% of tidal range changes in the entire North Sea including the divergent trend pattern along United Kingdom and German coastlines that reflects movement of the region’s semidiurnal amphidromic areas. By applying numerical and statistical analyses, we can assign a baroclinic (PC1) and a barotropic large‐scale signal (PC2), explaining a large part of the overall variance. A comparison between PC2 and tide gauge records along the European Atlantic coast, Iceland, and Canada shows significant correlations on time scales of less than 2 years, which points to an external and basin‐wide forcing mechanism. By contrast, PC1 dominates in the southern North Sea and originates, at least in part, from stratification changes in nearby shallow waters. In particular, from an analysis of observed density profiles, we suggest that an increased strength and duration of the summer pycnocline has stabilized the water column against turbulent dissipation and allowed for higher tidal elevations at the coast.

## Key Points

- 70 North Sea tide gauges evince contrasting trends in tidal range between the United Kingdom (−1.0 mm/yr) and the German Bight (3.3 mm/yr) since 1958
- We use principal component analysis (PCA) to separate local (e.g., building measures) from large‐scale effects of oceanographic origin
- The first PC explains 77% of variance in the German Bight and is linked to stability changes in shallow, seasonally stratified waters

## 1 Introduction

For thousands of years, tides have had a great influence on coastal areas globally and their residents. Today they play a critical role in influencing economic considerations, nautical safety, renewable energy schemes, assessments of land erosion, and the definition of geodetic datums (Haigh et al., 2020; Pugh & Woodworth, 2014). Tides not only control the navigability of some ports and sea routes, but also have a major influence on the intensity and timing of extreme sea levels during storm surges (e.g., Arns et al., 2020; Horsburgh & Wilson, 2007; Prandle & Wolf, 1978). Given their close connection to the periodic and predictable nature of astronomical variations, the amplitudes and phases of tidal constituents, and corresponding tidal water levels, are generally assumed to be constant on time scales over which basin geometry undergoes only minor changes (i.e., decades to centuries). However, Keller (1901) showed increased tidal amplitudes due to reflection and local resonance changes as a result of building measures such as weirs (e.g., in the Ems River). Similarly Doodson (1924) pointed to appreciable secular perturbations in the local tidal regimes of particular ports, weirs, and estuaries. More recently, the topic of changes in ocean tides has been revived and extended to the scales of shelves, basins and the global ocean—a development fueled by the digitization and publication of global data sets of tide gauge records, see P. Woodworth et al. (2017). In fact, statistically significant trends of tidal parameters of the order of a few percent (in relative terms) are now well documented around the world (e.g., Flick et al., 2003; Jay, 2009; Mawdsley et al., 2015; Ray, 2009; Talke & Jay, 2017; P. L. Woodworth et al., 1991). Fluctuations of similar magnitude and regional extent have been observed on interannual time scales (e.g., Devlin et al., 2014; Feng et al., 2015; Müller, 2011; Ray & Talke, 2019).

Despite this ample evidence of changes in tides in water level series, the forcing factors and spatial extent of secular and short‐term variability in tides remain uncertain. P. Woodworth (2010) succeeded in detecting coherent patterns of amplitude and phase trends in primary constituents along the North American coasts, but found less regional consistency in data from Asia, the Australian Seas or Europe. However, some spatially coherent changes could still be observed in smaller and well‐instrumented areas. A major problem identified by P. Woodworth (2010) is that small‐scale (often site‐specific) and large‐scale changes may occur simultaneously, thereby impeding research of the underlying physical processes. Over wider coastal sections, and at sites open to the sea, the effects of a rise in mean sea level (MSL) on tidal wave propagation explain only a fraction of the observed trends (Müller et al., 2011; Schindelegger et al., 2018). Accordingly, the assumption persists that other mechanisms—such as changes in stratification, turbulent dissipation, and variations in shoreline position or bed roughness—play major roles; see Haigh et al. (2020) for a review. The present consensus is that in many areas of the world a combination of different oceanographic processes may be at work. For instance, Ray and Talke (2019) suggest that the large secular changes of the lunar M_2_ tide in the Gulf of Maine could be caused by both sea level rise and persistent stratification changes. Yet, as implied above, any contributing mechanism will act on its own characteristic spatial and temporal scales, overlaying and possibly reinforcing other processes. This particularly applies to anthropogenic construction measures (e.g., building of dykes and tidal barriers) that can cause transient perturbations to the local tidal regime and affect adjacent stretches of coastline (Talke & Jay, 2020). Therefore, a major challenge is the separation of local effects and large‐scale changes and their subsequent attribution to certain forcing factors.

**Figure 1:**
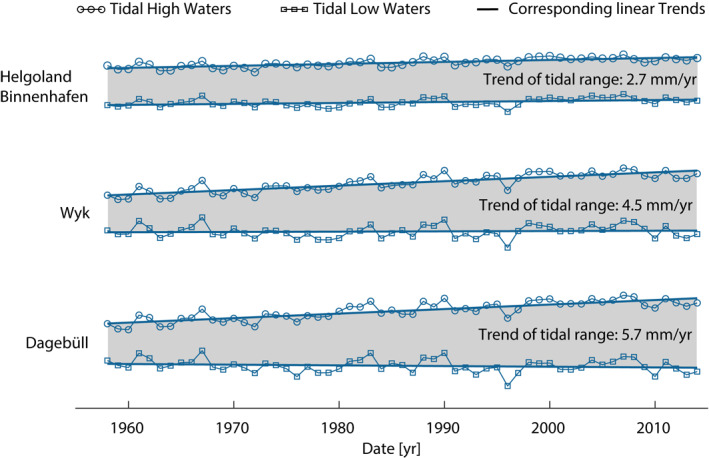
Time series of mean annual high and low tidal water levels for three exemplarily selected stations in the German Bight. For illustration purposes, all records are shown with different artificial vertical offsets. The increase in the tidal range is illustrated for the three sites as gray shaded areas between high and low water level time series.

**Figure 2:**
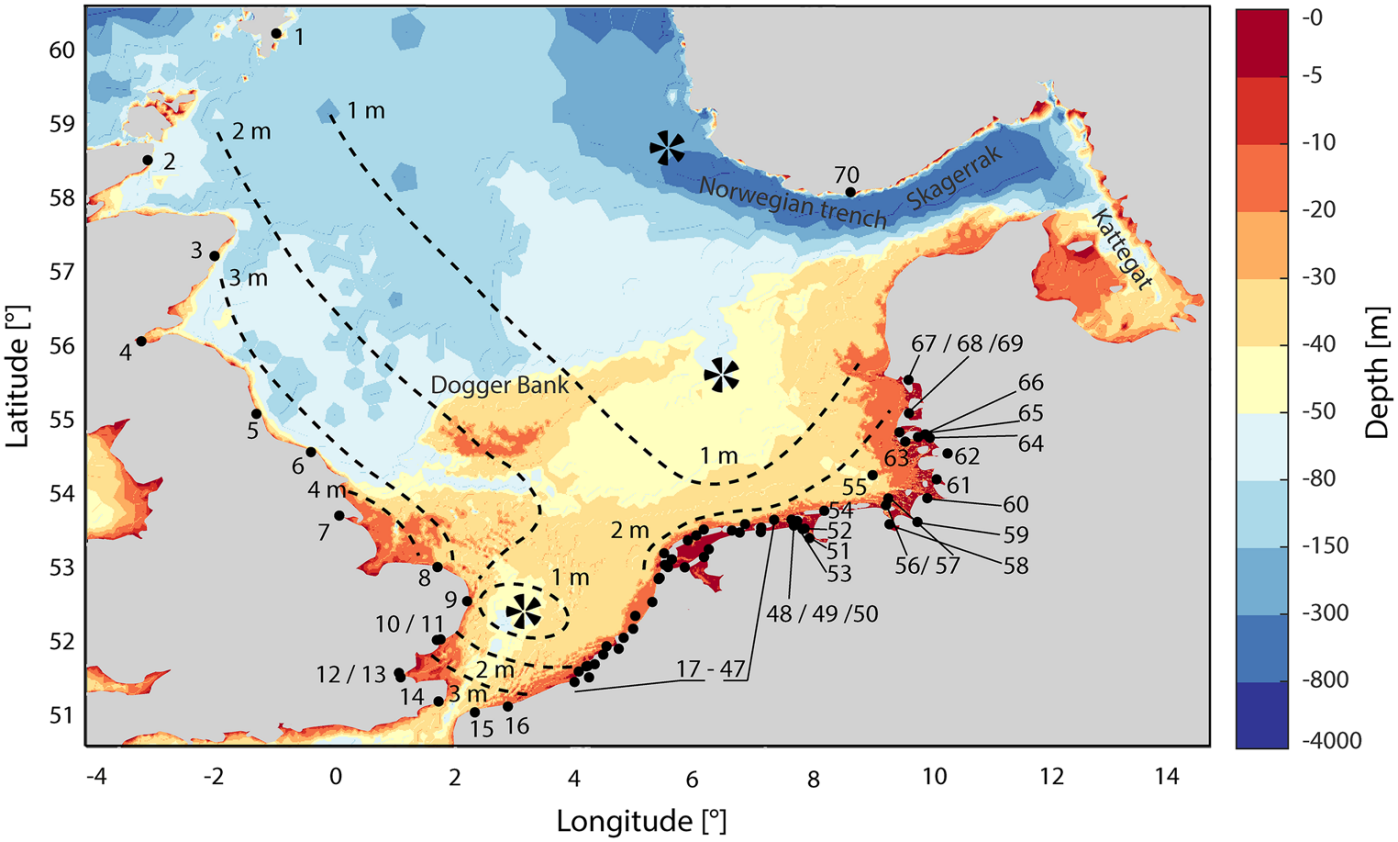
Bathymetry of the North Sea (Becker et al., 2009; Schrottke & Heyer, 2013). Also shown are the locations of tide gauges (black dots) used in this study including their respective numbering (see also Table 1). The black propellers indicate the location of the three semidiurnal amphidromic areas (including the amphidromic points for the M_2_ and S_2_ constituent) and the black dotted lines indicate contours of equal mean tidal range (Sündermann & Pohlmann, 2011).

Exceptional changes of tidal range in the German Bight have been documented as early as in Führböter & Jensen (1985) and are illustrated in Figure 1; see also Jensen (2020). Between 1958 and 2014, changes in tidal range amount to approximately 3% (e.g. Helgoland Binnenhafen, #55 in Figure 2/Table1) at some of the investigated tide gauges to more than 11% at others (e.g. Wyk auf Föhr, #65). The latter is equivalent to a trend of 5.7 mm/yr at Dagebüll (#66) and outpaces the simultaneous local (∼2 mm/yr, Dangendorf et al., 2015) and global MSL rise, which is approximately 3 mm/yr today (Dangendorf et al., 2019) and was 1.5 mm/yr between 1900 and 2012 (Oppenheimer et al., 2019). To our knowledge, this magnitude of tidal range change is one of the highest in the world, only exceeded by developments in the Gulf of Maine (Ray & Talke, 2019). It further seems that the overlap between local and large‐scale effects in the North Sea is particularly pronounced, possibly nurtured by the region’s character as a shelf sea with a tide generated in the Atlantic. Previous research (summarized in Jensen et al., 2014) has ruled out astronomical, large‐scale morphological, or tectonic causes (at least in the German Bight), but pointed to the generally non‐linear and non‐uniform behavior of water levels in the North Sea. To improve our understanding of these puzzling tidal range changes, we aim to address the following questions through systematic data analysis: (1) Are these changes on different time scales detected within the German Bight a localized phenomenon, or are they part of a larger‐scale development spreading over adjacent areas within or even outside the North Sea region? (2) Is it possible to separate and quantify large‐scale and small‐scale effects from observed records? (3) If (2) is the case; can we attribute physical causes to the observed changes?

**Table 1:** Name, Coordinates, Period and Coverage of the 70 Tide Gauges Used in This Study (See also Figure 2)

Tide gauge		Lon. [°]	Lat. [°]	Period [yr]	Cov. [–]	Tide gauge		Lon. [°]	Lat. [°]	Period [yr]	Cov. [–]
1	Lerwick	−1.14	60.16	1959–2011	0.83	36	Kornwerderzandbuiten	5.34	53.07	1958–2014	1.00
2	Wick	−3.09	58.44	1965–2014	0.79	37	Texel Noordzee	4.73	53.12	1990–2014	0.27
3	Aberdeen	−2.07	57.14	1958–2014	0.75	38	Harlingen	5.41	53.18	1958–2014	1.00
4	Leith	−3.18	55.99	1989–2014	0.39	39	Vlielandhaven	5.09	53.3	1958–2014	1.00
5	North Shields	−1.44	55.01	1962–2014	0.79	40	West‐Terschelling	5.22	53.36	1958–2014	1.00
6	Whitby	−0.61	54.49	1981–2014	0.55	41	TerschellingNoordzee	5.33	53.44	1989–2014	0.45
7	Immingham	−0.19	53.63	1958–2014	0.90	42	Nes	5.76	53.43	1971–2014	0.77
8	Cromer	1.30	52.93	1988–2014	0.45	43	Holwerd	5.88	53.4	1971–2014	0.54
9	Lowestoft	1.75	52.47	1964–2014	0.86	44	Wierumer‐gronden	5.96	53.52	1981–2014	0.60
10	Felixstowe	1.35	51.96	1982–2011	0.39	45	Lauwersoog	6.20	53.41	1971–2014	0.77
11	Harwich	1.29	51.95	1958–2014	0.29	46	Schiermonni‐koog	6.20	53.47	1966–2014	0.86
12	Southend	0.72	51.5	1958–1981	0.40	47	Huibertgat	6.40	53.57	1973–2014	0.74
13	Sheerness	0.74	51.44	1958–2013	0.64	48	BorkumFischerbalje	6.75	53.56	1963–2014	0.90
14	Dover	1.32	51.12	1958–2014	0.90	49	Borkum Südstrand	6.66	53.58	1958–2014	1.00
15	Calais	1.87	50.97	1965–2014	0.52	50	OudeWestereems	6.70	53.5	1981–1983	0.04
16	Dunkerque	2.37	51.05	1959–2014	0.68	51	EemshavenDoekegat	6.86	53.46	1983–1987	0.07
17	Cadzand	3.38	51.38	1971–2014	0.77	52	Eemshaven	6.83	53.45	1979–2014	0.63
18	Westkapelle	3.44	51.52	1958–2014	1.00	53	Delfzijl	6.93	53.33	1958–2014	1.00
19	Oostkapelle	3.56	51.59	1971–2014	0.77	54	NorderneyRiffgat und Hafen	7.16	53.7	1958–2014	1.00
20	Oranjezon	3.57	51.6	1979–1987	0.14	55	HelgolandBinnenhafen	7.89	54.18	1958–2014	1.00
21	Roompot‐buiten	3.68	51.62	1972–1974	0.04	56	LT Alte Weser – Roter Sand	8.13	53.86	1958–2014	1.00
22	Brouwers‐havenscheGat08	3.81	51.75	1987–2014	0.49	57	WilhelmshavenAlter Vorhafen	8.15	53.51	1958–2014	1.00
23	Haringvliet10	3.86	51.86	1980–2014	0.61	58	Bremerhaven	8.57	53.55	1958–2014	1.00
24	Haringvliets‐luizenbuiten	4.04	51.83	1982–2014	0.54	59	Mellumplate	8.09	53.77	1963–2014	0.91
25	Hoek van Holland	4.12	51.98	1972–1987	0.26	60	CuxhavenSteubenhöft	8.72	53.87	1958–2014	1.00
26	Scheveningen	4.26	52.1	1958–2014	1.00	61	Büsum	8.86	54.12	1958–2014	1.00
27	Noordwijk‐meetpost	4.30	52.27	1961–2005	0.76	62	Husum	9.02	54.47	1958–2014	1.00
28	Ijmuiden‐buitenhaven	4.55	52.46	1984–2006	0.36	63	Wittdün	8.38	54.63	1958–2014	1.00
29	Pettenzuid	4.65	52.77	1981–2014	0.60	64	Schlüttsiel	8.76	54.68	1961–2014	0.94
30	Petten	4.66	52.79	1978–2014	0.61	65	Wyk auf Föhr	8.58	54.69	1958–2014	1.00
31	Den Helder	4.74	52.96	1971–1974	0.05	66	Dagebüll	8.69	54.73	1958–2014	1.00
32	Oostoever	4.79	52.93	1958–2014	1.00	67	Hörnum	8.30	54.76	1958–2014	1.00
33	DenOeverbuiten	5.05	52.93	1971–1981	0.15	68	List	8.44	55.02	1958–2014	1.00
34	Oudeschild	4.85	53.04	1958–2014	1.00	69	Esbjerg	8.43	55.47	1958–2014	0.92
35	Vlissingen	3.60	51.44	1958–2014	1.00	70	Tregde	7.55	58.01	1958–2014	0.40

Below, we first discuss geographic and oceanographic characteristics that are fundamental to the understanding of the tidal regime in the North Sea, the available database, its limitations, and major processing steps (Section 2). Section 3 introduces the analytical methods of Ordinary Kriging, which is here mainly used for gap‐filling as the subsequent PCA requires complete time series. The results of our analyses are described extensively in Section 4. To answer the abovementioned research questions, we start our analyses with the detection of observed changes in the tidal range at individual sites. In a second step, we apply a PCA to identify modes of variability common to all (or the majority of) sites and to distinguish them from local anomalies. In a last step we analyze potential causes and drivers of the observed changes. The paper concludes with a summary and additional remarks in Section 5.

## 2 Study Area and Data Basis

### 2.1 Study Area

The North Sea is one of the largest shelf seas on Earth with a size of about 575,300 km^2^ (Huthnance, 1991). Counted counter‐clockwise, its margins comprise coastal sections of the United Kingdom, France, Belgium, the Netherlands, Germany, Denmark, and the south of Norway (Figure 2). The North Sea is connected to the North Atlantic via a large inlet between Scotland and Norway in the north and a narrow opening through the English Channel in the southwest and it opens to the Baltic Sea in the east. Water depths in the North Sea are on average 90 m but vary greatly, generally increasing from south to north. While the southern parts are often shallower than 40 m with lowest depths in the German Bight, they increase to about 300 m at the continental shelf toward the Norwegian Trench and toward the entry into the Norwegian Sea in the northwest. There are also extensive shallow water regions off the south‐eastern coast of the United Kingdom known as the Dogger Bank complex, with their western part extending to the coasts of Norfolk and Suffolk (Quante & Colijn, 2016).

The tidal regime in most parts of the North Sea is strongly influenced by the astronomical, mainly semidiurnal, tides entering the basin from the Atlantic. The greater part of these oscillations enters between the Shetlands and Scottish mainland and a smaller part through the English Channel. They travel counter‐clockwise through the entire North Sea basin as Kelvin waves. The entry times of the tidal high and low waters are therefore shifted relative to each other according to the celerity of the tidal wave. This physical setting results in three amphidromic points, one close to the English Channel, one off the coast of Norway, and one central in the North Sea basin (Proudman & Doodson, 1924). Since the North Sea’s basin shape is close to the resonance frequency in the semidiurnal spectral band, the superposition of the principal lunar and solar tides M_2_ and S_2_ leads to a significant spring neap cycle. These two constituents cause a potential tidal range between 1 and 5 m (Quante & Colijn, 2016). Accordingly, the tidal regime of the North Sea can be classified as macrotidal (>4 m), mesotidal (2–4 m) and microtidal (<2 m) (Haigh, 2017), with the actual tidal range being strongly influenced by local factors. For example, the mean spring tidal range at the east coast of the United Kingdom varies between 3.60 m (Aberdeen) and 6.20 m (Immingham) (Horsburgh & Wilson, 2007). The mean tidal range in the data set used below is about 3.40 m in the UK and the English Channel, 1.98 m at the Dutch west coast, 2.33 m at the Dutch north coast, and 2.82 in the German Bight.

### 2.2 Data

**Figure 3:**
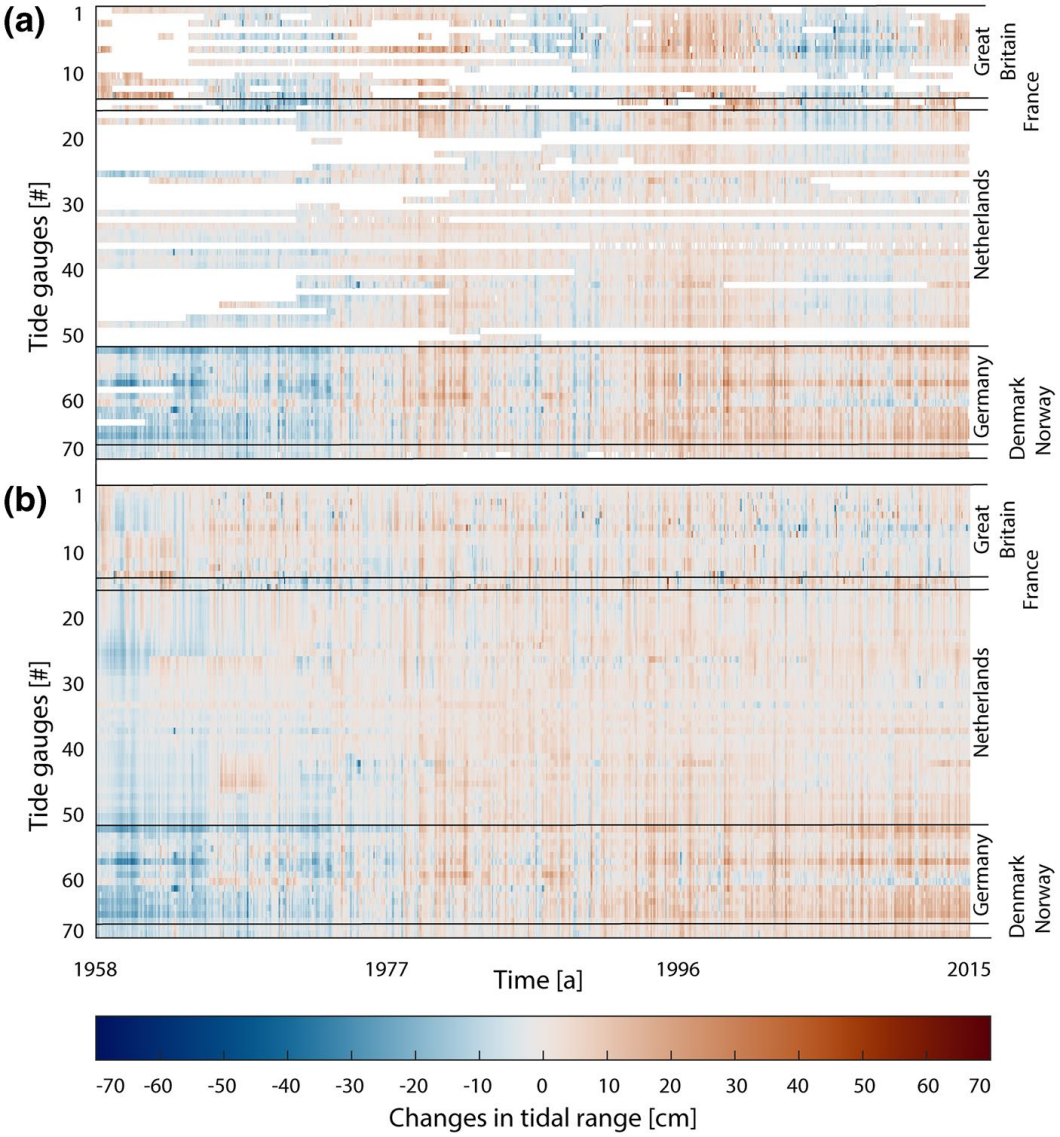
Changes in tidal range before (a) and after (b) applying ordinary kriging and removing the nodal cycle.

Time series of water level from 70 available tide gauges around the North Sea basin were collected from various sources. Data from Global Extreme Sea Level Analysis, GESLA Version 2 (GESLA; P. Woodworth et al. 2017), Open Earth (Deltares) and the responsible German authorities (Wasser‐und Schifffahrtsverwaltung des Bundes via the portals of the associated Central Data Management, ZDM) were used. The available time series vary considerably in length and completeness. The earliest measurements in the form of tidal high and low water readings are from 1843 (Cuxhaven Steubenhöft, Germany, #60), while on the Dutch coast, data from some stations have only been digitally available since the 1980s. High‐resolution data sets with an equidistant sampling between 1 and 60 min were used as well as time series of tidal high and low water. We excluded equidistant time series with a resolution lower than 60 min, as supplemental analyses have shown that they insufficiently describe the height and timing of individual tidal high and low waters. The tidal range was calculated as the difference between each tidal high water and the mean of the two surrounding tidal low waters, according to the German standard (DIN 1994‐3, 1994). From those, we calculated monthly averages and removed the mean seasonal cycle, as we are mainly interested in longer‐term changes. Considering the 18.6‐year nodal cycle and the end of numerous water level series in December 2014, we adopt an analysis period from January 1958 to December 2014, approximately 3 nodal cycles. Tide gauges known to be located near to weir installations or in rivers were excluded, as these are at least partially separated from the oscillation system of the North Sea. Seven time series of tidal range remained in the data set, forming the basis for our investigations (Table 1, Figures 2 and 3). Acknowledging the counter‐clockwise propagation direction of the tidal wave, the tide gauges used in this study are counted by starting at Lerwick (Shetland Islands) and ending at Tregde (Norway). The average completeness of the stations is 64% in the United Kingdom, 65% in the Netherlands, and around 88% in Germany.

The statistical analyses and procedures (Ordinary Kriging, Trend analysis, PCA) carried out here are based exclusively on the tide gauge records named in Table 1. In Section 4.4, the possible correlation between the records from the North Sea and the adjacent North Atlantic is examined. For this purpose, 24 additional North Atlantic tide gauges from the GESLA data set were used (Port‐aux‐Basques, Argentia, Saint John, Reykjavik, Cascais, Vigo, La Coruna, Santander, Saint Jean de Luz, Bayonne Boucau, Port Bloc, Les Sables D’Olonne, Saint Gildas, Port Tudy, Brest, Le Conquet, Newlyn, Roscoff, Devonport, Saint‐Malo, Cherbourg, Le Havre, Newhaven and Dieppe, P. Woodworth et al., 2017).

## 3 Methodology

In addition to the procedures explained in the following sections, linear trend analysis, harmonic analysis of tidal constituents, and wavelet coherence analysis were carried out to characterize multiple feature of the tide gauge records in the North Sea. Any significance statements made throughout the manuscript are based on a 95% confidence level. We calculated linear trends using ordinary least squares regression and assessed their significance by considering normally distributed but serially correlated residuals following an autoregressive process of the order 1 (e.g., Mawdsley & Haigh, 2016). Annual amplitudes for the leading constituents were determined by a harmonic analysis using the MATLAB toolbox U‐Tide (Codiga, 2011) and the wavelet analyses were conducted with the MATLAB package of Grinsted et al. (2004). None of these methods are explained here in detail due to their general recognition and widespread use.

Furthermore, an existing two‐dimensional, depth‐averaged barotropic tide, and surge model of the North Sea and the adjacent Atlantic Ocean (approximately from 48°N to 62°N and from 12°W to the transition between North Sea and Baltic Sea) developed by Arns et al. (2015a, 2015b) was used to simulate total water levels from 1958 to 2014. The original version of this model, based on the Danish Hydraulic Institute's (DHI) Mike21 FM (flexible mesh) model suite from 2014, was updated to the 2019 version for our analyses. At the open boundaries, we used the Technical University of Denmark DTU10 ocean tide model (Cheng & Andersen, 2010) as tidal input, and the MSL reconstructions of Wahl et al. (2013) were employed in order to incorporate the effects of rise in MSL. The entire model domain was forced with the 20th Century Reanalysis (20CR) data set of the US National Oceanic & Atmospheric Administration (NOAA) and the Cooperative Institute for Research in Environmental Sciences (CIRES) to describe the meteorologically induced effects on water levels (Compo et al., 2011).

### 3.1 Kriging

Kriging (also Gaussian process regression) is a geostatistical method to interpolate missing values based on information stemming from neighboring stations (i.e. their covariance matrix). It is here mainly used for gap‐filling as the following Principal Component Analysis (PCA) requires complete time series. Originally developed in the 1950s for mining purposes (Krige, 1951), this method has been used increasingly in other areas including the analysis and interpretation of incomplete surface air temperature fields (Rigor et al., 2000; Rohde et al., 2013). In general, Kriging is a linear interpolation procedure. Missing values are determined according to a given covariance matrix, which is calculated from the existing observations (Cressie, 1990). Kriging provides some important advantages over other interpolation procedures. The interpolated values change smoothly and always pass through the observed values at the sample points. Problems related to the accretion of measurement points are avoided by considering the statistical distances between the neighbors used in the interpolation of a certain value, which means that the spatial variance is taken into account. If clustering occurs in a region, the weights of the affected sample points are reduced by including the density. In sparse regions, only the distance is considered. The procedure can be summarized with the formula

(1) $${\hat{Z}}_{\left(x_{0}\right)}=\;\left[w_{1}\;w_{2}\ldots \;w_{n-1}\;w_{n}\;\right]\cdot \left[\begin{array}{l} z_{1}\\ z_{2}\\ \begin{array}{l} \vdots \\ \begin{array}{l} z_{n-1}\\ z_{n} \end{array} \end{array} \end{array}\right]=\sum_{i=1}^{n}w_{i}\left(x_{0}\right)\times Z\left(x_{i}\right),$$

where $\hat{Z}$ is the query value at the unobserved location *x*_0_ and *i* = 1 … *n* represents a running index over n observations. $\hat{Z}$ is computed from a linear combination of all observed values *z*_*i*_ = *Z*(*x*_*i*_), which are weighted by the parameter w according to distance and density. A special property of the Kriging procedure is the convergence of interpolated values to the mean value of their region with increasing distance to the available samples. That is why Kriging estimates at query points tend to be conservative (Cowtan & Way, 2014). In keeping with this characteristic, the general tidal range behavior worked out later in Section 4.1 is also valid when the Kriging step is omitted.

**Figure 4:**
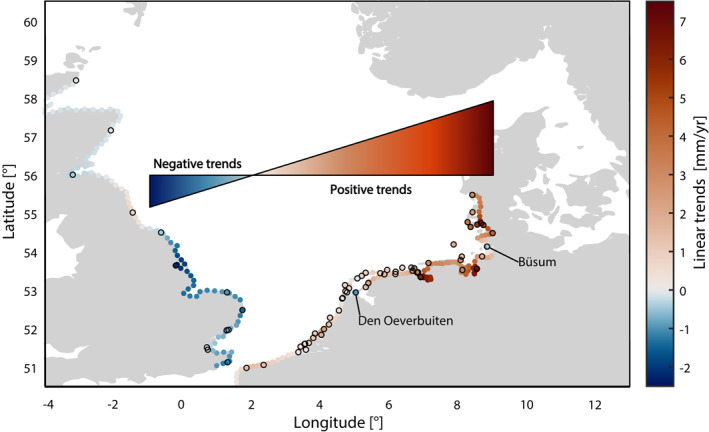
Linear trends of tidal range between 1958 and 2014. Trends at measured sites are shown as dots with a black edge. Dots in between stations are based on Kriging.

We use Kriging for two different purposes. First, the temporal gaps in the tidal range data (Section 2.2) were closed for each monthly time step in the investigation period. Figure 3a illustrates that this is a relevant issue in the Netherlands, in particular before 1970, while in the UK data gaps occur before 1990. Second, additional data points along the coastline of the North Sea were interpolated, allowing us not only to analyze the temporal evolution of each station series in terms of a linear trend but also the spatial structure of these trends (Figure 4). For both applications, we use the Ordinary Kriging algorithm of Schwanghart (2020). Note also that in transitioning from Figure 3a to Figure 3b, the nodal cycle (with peaks for semidiurnal M_2_ in the years 1977, 1996, and 2015) was removed.

### 3.2 Principal Component Analysis

Principal Component Analysis (PCA), a method of multivariate statistics, is used to structure and simplify extensive data sets by approximating a large number of statistical variables with a smaller number of significant, non‐correlated (orthogonal) linear combinations. If *x* is a vector with *n* random variables, first a linear function *f*_1_(*x*)—dependent on constant coefficients *c*_1*i*_—is determined by calculating the eigenvector from the spatially weighted covariance matrix of *x*. Then *f*_1_(*x*) represents the largest possible overall variance of all variables in *x*

(2) $$f_{1\left(x\right)}=c_{11}\cdot x_{1}+c_{12}\cdot x_{2}+\cdots c_{1n-1}\cdot x_{n-1}+c_{1n}\cdot x_{n}=\overset{n}{\underset{i=1}{\sum }}c_{1i}\cdot x_{1i}$$

This decomposition process is repeated for a function *f*_2(*x*)_, which is uncorrelated with *f*_1(*x*)_ and describes the largest possible amount of the remaining variance. It is possible to find n such functions, but the purpose is usually to explain as much variance as possible with significantly fewer functions *f*_*i*(*x*)_, known as Principal Components (PCs) (Jolliffe, 2002). Therefore, the PC of a temporally and/or spatially varying physical process represents orthogonal spatial patterns, in which the data variance is concentrated. Using the leading PC, an approximate reconstruction of the observed variable can be generated. This type of analysis is often used in Earth system sciences to identify spatial and temporal patterns of climate oscillations (e.g., Barnston and Livezey, 1987; Berx & Pain, 2017; Häkkinen & Rhines, 2004).

In this study, we apply PCA to the entire monthly de‐seasoned tidal range data set from the 70 sites (Figure 2), whose gaps were previously filled through Ordinary Kriging. If there are indeed large‐scale signals affecting the tidal range in the North Sea, they should appear as a coherent pattern at multiple sites, and therefore be visible in the leading PCs. By contrast, spatially confined (“small‐scale”) anomalies in tidal range will be shifted into the higher PCs, as these can only be responsible for a small part of the overall variance. Such shifting includes not only the response of the local tidal system to, for instance, anthropogenic construction measures but also to changes in bathymetry or morphology. Local effects can explain more variance than large‐scale effects at individual sites or small subsets, but never for the entire data set. It is therefore important to consider the explained variance of the PCs at each tide gauge individually to ensure that large‐scale effects with a very small influence on the overall variance are retained. With this approach, the PCA enables us not only to attribute tidal range changes to small‐scale and large‐scale effects, but also to calculate the spatial extent and the temporal development of patterns that might reflect important environmental factors.

## 4 Results and Discussion

### 4.1 Trends of Tidal Range and Tidal Constituents

To address the three research questions defined in the introduction, we first map the spatial extent of the long‐term changes in tidal range in the study area. We start our analysis by calculating linear trends for each individual record over a common period between 1958 and 2014 and map them in Figure 4. In this step of the analysis, the time series of Lerwick (Shetland Islands) and Tregde (Norway) were omitted, since both are the only available tide gauges within large areas and, therefore, there is insufficient data density for use by the Kriging algorithm. We identify a variety of trends with a particularly pronounced spread in the southern parts of the basin. While there are no significant trends at the north‐eastern coast of the United Kingdom, negative trends occur further south between Immingham and Dover. Here, six of eight stations show significant negative trends while the remaining two do not differ significantly from zero. In this area, Immingham shows the largest negative and statistically significant trend (−2.3 ± 0.5 mm/yr) of all sites, while the smallest negative trend of −0.7 ± 0.3 mm/yr is found in Felixstowe. The mean value for all tide gauges in this area is 1.0 mm/yr. In contrast, trends turn positive on the continental side of the English Channel and the European West Coast. Our assessment reveals increasing trends following the coastlines of France (1.3 ± 0.4 mm/yr at Dunkerque), Belgium and the western Netherlands up to the tide gauge at Huibertgat (0.8 ± 0.2 mm/yr), near to the German‐Dutch border. On average, trends along the European West Coast are 0.8 mm/yr. Hereafter, sharp trend increases are found within a short distance, reaching values of more than 7 mm/yr in the German Bight area. Here, the average trend in tidal range amounts to 3.3 mm/yr (Table 2). Local changes affect some tide gauges like Den Oeverbuiten (Netherlands) or Büsum (Germany), which at first sight seem to contradict this spatial pattern. We suggest that these local exceptions are mainly caused by anthropogenic interventions such as the building of the Afsluitdijk at Den Oeverbuiten or dredging and dike constructions near to Büsum, which coincide with anomalies in the local tidal range series. From the aforementioned findings, we conclude that widespread and statistically significant secular changes in tidal range occurred around large parts of the southern North Sea between 1958 and 2014, although locally interrupted by opposing signals at individual sites. Furthermore, we note contrasting and dipole‐like trends along south‐western (significant negative values) and south‐eastern margins of the North Sea (significant positive values). It remains to be critically noted that the changes in the tidal range at some individual tide gauges could also be instrumental. However, due to the large‐scale and the spatial homogeneity of the patterns, this cannot be causal for the overall picture.

**Table 2:** Measured and Reconstructed Trends in Tidal Range and Explained Variance of the Different Regions

Location		Mean linear trends [mm/yr]		Explained variance [%]		
Region	Tide gauges	Measured	Reconstructed PC1 and PC2	PC1	PC2	Remaining PCs (local)
Southwestern Coast of GB	Immingham to Dover	−1.0	−1.0	3	58	39
European West Coast	Calais to Huibertgat	0.8	1.0	45	10	45
North Coast of the Netherlands and German Bight	Oude Westereems to Esbjerg	3.3	3.5	77	3	20

PC, principal components.

**Figure 5:**
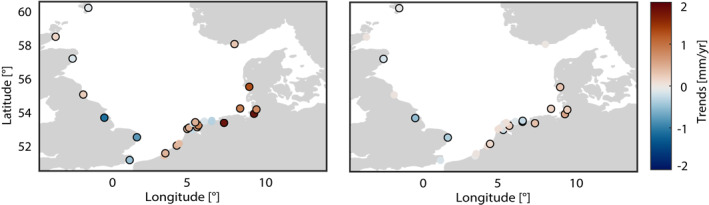
(a) Linear trends of the M_2_ and (b) S_2_ tidal constituents between 1958 and 2014 (significant trends outlined).

The identified dipole‐like trend pattern has its node approximately at the longitude of the English Channel (Figure 4) and suggests a westward displacement of the main low amplitude areas (including amphidromic points of M_2_ and S_2_) located in the central North Sea and near the English Channel (Figure 2). To obtain further indications of such a shift, we have performed a harmonic analysis to determine the main semi‐diurnal M_2_ and S_2_ tidal constituents, which make the largest contributions to the tides in the North Sea. Since high‐resolution hourly time series with a coverage of at least 75% between 1958 and 2014 are required for a tidal analysis, only a subset of 28 tide gauge records is appropriate for our assessment. The available database is thus reduced and fewer stations show significant trends (20 for M_2_, 14 for S_2_). Nevertheless, the overall findings (Figure 5) are similar to the assessment focusing on tidal ranges highlighted in Figure 4; that is for both constituents (though with larger magnitude for M_2_), negative trends occur in the southeast of the UK and the highest positive trends are found in the German Bight area. A displacement of the M_2_ and S_2_ amphidromic point is, therefore, also implicated.

The observed changes in the tidal range can be considered in the context of the elaborations of Taylor (1922) on amphidromic systems. Based on simple analytical solutions, Taylor demonstrated an altered propagation speed due to increased water depth, leading to a shift of the amphidromic point toward the open boundary in a semi‐enclosed basin. As a result, the tidal range at the opposite (dissipative) end of the basin increases. In our case, this statement implies a shift of the amphidromic points toward the north, seemingly contradicting the changes (i.e., an east‐west shift) observed here. However, as pointed out in Haigh et al. (2020), increasing the tidal range and thus the tidal currents at the dissipative end could lead to a higher frictional energy loss. This would cause a leftward deflection of the tidal wave and the amphidromic point, see Figure 5 in Haigh et al. (2020). For the North Sea, MSL rise and an increased frictional dissipation would ultimately shift the amphidromic point toward the west, reduce of the tidal range on the left side of Figure 4 (the east coast of the UK) and an increase on the right side of the basin (the German Bight). This argument is supported by several numerical modeling efforts (Idier et al., 2017; Pickering et al., 2012; Schindelegger et al., 2018), in which the impact of large (up to 10 m) MSL increases on leading constituents (mainly M_2_) were investigated. Complementary to our empirical assessment, they all detected (at least qualitatively) similar patterns as shown in Figures 4 and 5. However, closer examination also reveals some discrepancies and the model results do not correspond exactly to the measured data. For instance, both Pickering et al. (2012) and Schindelegger et al. (2018) predict an increase in M_2_ amplitude in the southwestern part of the North Sea, between Suffolk/Essex and the Netherlands, while we detect negative trends in Suffolk/Essex and positive trends in the Netherlands. In‐depth studies of the influence of Sea level rise on European Shelf tides (Idier et al., 2017; Pelling and Green, 2014) point to sensitivities of the tidal response to the magnitude of Sea level rise and whether or not low‐lying land is inundated in the numerical simulation (flooding or no‐flooding). In addition to these two extreme cases of shoreline treatment, Pelling and Green (2014) investigated the M_2_ response to partial flooding, roughly based on the actually existing protective structures. This last option provides the greatest agreement with our results, but again does not reflect the negative trends in the South East UK. In fact, the tide around Suffolk/Essex exhibits little sensitivity to the shoreline scenario (Idier et al., 2017). More to the point, the assumption of no‐flooding seems to be plausible in the areas of the greatest changes (German Bight, northern parts of the Netherlands) and here the results agree with all existing modeling studies. No final assessment can thus be made here as to whether and which models are most consistent with the observations. In this context, effects may be at work that are not included in numerical models so far. As Arns et al. (2015a) point out, various non‐linear relationships between the individual parameters in marginal seas are of particular importance, especially the dynamic response of the sea surface to meteorological forcing (see also Arns et al., 2020). In addition, time‐varying bed roughness and bottom friction coefficients (Rasquin et al., 2020) and changes in turbulent dissipation with stratification (Müller 2012) may play a role.

### 4.2 Principal Components and Large‐Scale Effects

**Figure 6:**
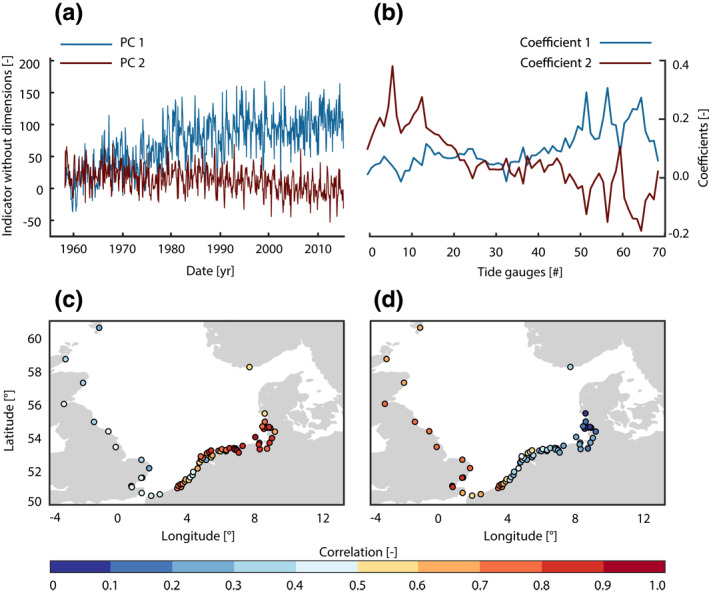
Results of the PCA. (a) Shown are time series of PC1, and PC2 and (b) their corresponding spatial patterns. Panels (c) and (d) map the correlations between observations and PC1 (c) and PC2 (d) for each site. PCA, principal component analysis.

**Figure 7:**
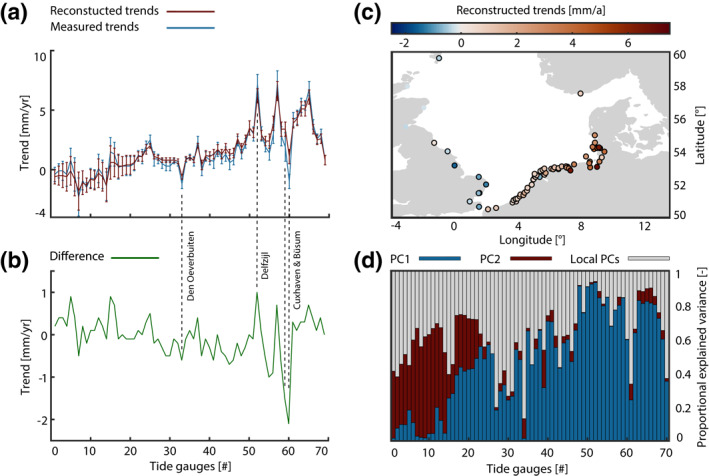
Linear trends in tidal range with 95% significance intervals from measurements (blue) and the reconstruction (red) based on PC1 and PC2, with the respective difference shown in (b). (c) Spatial distribution of the linear trends from the reconstruction (significant trends outlined) and (d) explained variance of the two PCs as share of the total variance.

Our results of the linear trend analysis point toward a distinct spatial pattern that is occasionally interrupted by diverging trends at individual locations. To further distinguish between the large‐ and small‐scale effects of tidal range changes—comprising both trends and short‐term variability—we apply PCA (Figure 6). The first two PCs, which are presented in Figure 6, explain about 69% of the total variance in the entire data set (PC1: 55%, PC2: 14%), while each of the remaining 68 PCs contributes between 0.01% and 4%. Additionally, no other PC represents significant parts of the variance at a larger number of tide gauges and is therefore rather local in character. This indeed suggests that the two leading PCs reflect coherent large‐scale effects, while local effects through anthropogenic interventions are retained in the reminder of the lower PCs. The amount of these percentages depends to some extent on the spatial distribution of the tide gauges, making it necessary to consider the PCA results at each tide gauge (Figures 6c,d, 7d). PC1 describes an increase in tidal range over time, as evident from its positive slope and the consistently positive values of the associated coefficients at all sites (Figure 6a). The magnitudes of the coefficients reveal that the signal represented by PC1 increases as one travels counterclockwise throughout the basin reaching its strongest expression in the German Bight. PC2 exhibits a negative trend and is most pronounced in the area of the southeastern coast of the UK. The coefficients of PC2 change sign from positive values along the UK coast to negative values in the area of the German Bight (Figure 6b). Similar to the trends of measured tidal range (Figure 4), a dipole‐like temporal evolution with a node in the area of the English Channel is detected. In general, PC1 accounts for the increase in tidal range in the German Bight and PC2 represents the decrease in tidal range at the south‐eastern coast of the UK. This contrast is also reflected in the correlation coefficients of the first two PCs with the measured tidal range changes (a metric that is mostly influenced by inter‐ and intra‐annual variability). Figure 6c shows moderate but significant correlations of 0.3–0.5 for PC1 at the south‐western boundary of the North Sea and displays the highest values (∼0.9) in the area of the German Bight. A contrasting picture emerges for PC2. In the area of the German Bight, correlations with tidal range changes are non‐significant and close to zero but almost consistently above 0.7 and significant in the United Kingdom (Figure 6d).

These patterns are also confirmed when considering the explained variance for particular clusters of tide gauges. Along southeastern United Kingdom coastlines, where negative trends are found, the explained variance of PC1 amounts to only 3%, while PC2 explains about 58% (Table 2). In the Netherlands, the mean explained variance for PC1 is 45% and only 10% for PC2. The contribution of the second mode drops to 3% in the German Bight, whereas PC1 explains 77% of the variance on average. This spatially reversing pattern is also detectable in the coefficients for PC1 and PC2 (Figure 6b), just as in the linear trends of the tidal range observations. Apparently, PC1 with its positive slope is more pronounced in the area of the German Bight, whereas PC2 (negative slope) dominates in the southeast of the United Kingdom. This indicates different underlying physical mechanisms for these large‐scale signals.

### 4.3 Impacts on Local Tidal Range

After identifying two large‐scale patterns relevant at the majority of tide gauge records in the North Sea, we next ask whether we also can identify small‐scale effects using the residual signal after removing the linearly regressed PC1 and PC2 at individual sites. Figure 7d shows the explained variances and indicates that alongside the described contrast between PC1 and PC2, local influences play a major role in some cases. Especially noticeable are again tide gauges Den Overbuiten (Netherlands, #33) and Büsum (Germany, #60) due to their high percentage of local effects. For example, PC3 (explained overall variance: 4%) captures more than 50% of the variance at Büsum and around 30% at Cuxhaven (Germany, #59). This anomaly is reflected in the comparison of the measured trends with those from re‐synthesizing PC1 and PC2 (Figure 7a). The confidence bounds show clear overlaps for most cases, but not at tide gauges Den Overbuiten, Büsum, and Cuxhaven. The local characteristics are sufficiently pronounced to overshadow the large‐scale signals, which is also evident from the difference between measured and reconstructed trends in Figure 7b. In this plot, the 1.0 mm/yr residual at Delfzijl (Netherlands, #52) stands out, too. This difference can also be traced back to significant local effects, most likely caused by the deepening of the outer areas of the Ems (Hollebrandse, 2005). Hence, local effects have a very large influence on the explained variance at individual sites. However, the general trends at most gauges can be qualitatively and quantitatively reproduced by PC1 and PC2. Figure 7c underlines this statement by a spatial map of the reconstructed trends, again highlighting the dipole‐like pattern between UK and German Bight sites. Comparing with the estimates in Section 4.1, the mean trend of tidal range synthesized from PC1 and PC2 at the southwest coast of the United Kingdom is −1.0 mm/yr, just like the measured trend (Table 2). Similar findings apply to the European west coast, where an average reconstructed trend of 1.0 mm/yr is achieved compared to 0.8 mm/yr from the in situ data. Local effects increase the tidal range by 0.2 mm/yr on average. In the German Bight, the trend from our reconstruction is 3.5 mm/yr, overshooting the measured trend by 0.2 mm/yr. Hence, we conclude that the opposing trends between the United Kingdom and the German Bight are largely controlled by the physical processes driving PC1 and PC2.

### 4.4 Identifying Physical Causes

**Figure 8:**
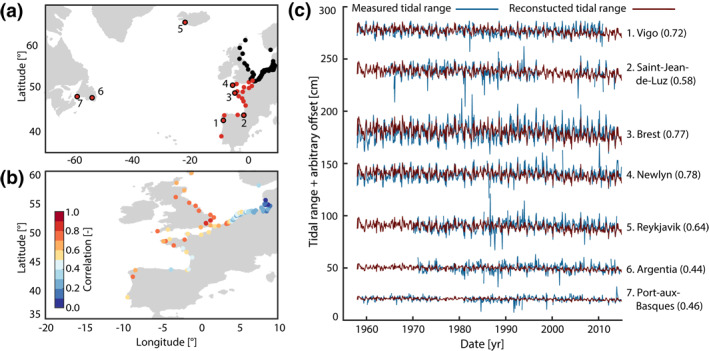
(a) Extended network of tide gauges with additional stations shown in red, (b) correlations of all tide gauges (except 5–7) with PC2 and (c) comparison between measured and reconstructed values of tidal range at the newly added tide Gauges 1–7. The reconstruction in (c) is based on PC2, and the numbers in parentheses indicate the respective correlation.

The PCA suggests two modes of variability (Figure 6a) that appear coherently at the investigated sites in the North Sea. Now the question naturally arises whether these signals are produced within or outside the basin. If the former is the case, then the corresponding PCs should show no correlations to tide gauge records from the adjacent North Atlantic, while an external forcing would possibly provide some sort of coherence with those records. Therefore, PC1 and PC2 generated from tide gauges inside the North Sea basin were compared with selected tide gauges from outside the North Sea basin in the North Atlantic, which were not included in the PCA. To that end, the additional 24 North Atlantic tide gauges from the GESLA data set described at the end of Section 2.2 were used. No coherence is found for PC1 and we therefore conclude that it is produced within the basin, which will be addressed later. The opposite applies to PC2. A comparison between PC2 and available tide gauge records along the European Atlantic coast, Iceland and Canada is shown in Figure 8. Figure 8c indeed documents high and significant correlations of about 0.7 on average between PC2 (calculated exclusively on the basis of North Sea data set) and Atlantic tide gauge records spanning the region from the English Channel southward to Spain. Moreover, there are significant correlations of 0.64 in the north (Reykjavik, Iceland), and even in the Northwest Atlantic (still reaching 0.46 in Port‐aux‐Basques, Newfoundland) (Figure 8a, c). Further south toward the Gulf of Maine, these correlations disappear (not shown). A supplemental wavelet analysis (not shown) reveals that the common oscillations between PC2 and the measured tidal range changes mainly occur on time scales from 6 to 24 months with particularly high coherence at around 12 months. We interpret this finding as an indication for a common high‐frequency signal in the North Atlantic of unknown origin, causing widespread changes in tidal range.

In order to narrow down the possible causes for the PC2 signal, outputs from the barotropic shallow‐water model run by Arns et al. (2015a, 2015b) over the period 1958 to 2014 were used. To facilitate a rigorous comparison with our in situ data, simulated time series at the locations of the 70 tide gauge stations were extracted. A PCA revealed that the PC2 pattern is represented well in the simulated data. We find similarly high correlations between the model‐based PC and the observations of the Atlantic tide gauges. While the mean correlation of the European tide gauge records (Figure 8b) with North Sea PC2 from observations is 0.70 (*p* < 0.05), it is only marginally lower with the barotropic model outputs (*r* = 0.66). If the simulated signal is removed from the model, the correlation becomes insignificant and even disappears at most sites. In consequence, PC2 must be driven by a process initially included into the boundary conditions from the numerical model. Since we have used a barotropic formulation without buoyancy forcing and thermodynamic calculations, we can further infer a purely barotropic relationship. Amongst the possible relevant factors, the tidal input to the model can safely be neglected. The DTU10 tide model consists of 10 tidal constituents, stationary in time and modulated only by the 18.6‐years nodal cycle. The high correlations on the east coast of the United Kingdom and in the North Atlantic are unrelated to this forcing, since purely tide‐induced changes would be periodic and present in the remaining parts of the North Sea.

The effects of bottom friction are more involved, but some simple geometric considerations are instructive. As the tidal wave enters the extensive shallow water areas of the southern North Sea, energy losses due to friction become dominant, yet the influence of PC2 is increasingly attenuated in the direction of propagation (Figure 6d). This discrepancy suggests that frictional effects do not represent the physical cause of PC2, although they might play a role in suppressing the magnitude of PC2 in the highly dissipative eastern North Sea region. As our simulations were performed with an invariant bathymetry and no changes to friction parameters, sea level rise and meteorological forcing remain as possible causes. We therefore analyzed correlations between PC2 and these factors (MSL rise, atmospheric pressure loading, wind velocities, and directions) but could not detect a clear and significant linear relationship. In this context, Arns et al. (2015a) already referred to the numerous non‐linear relationships between the individual parameters in marginal seas. Specifically, the nonlinear interaction between tide and sea level rise as well as the dynamic response of the sea surface to meteorological forcing are important (see also Arns et al., 2020). Further analyses, in particular sensitivity studies taking into account altered tidal boundary conditions and time variable friction coefficients, will perhaps allow for a final identification of the ultimate driving factors (e.g., Rasquin et al., 2020).

While the signal of PC2 is reproducible, PC1 cannot be detected in the simulated data, which means PC1 is absent in the barotropic model. At the beginning of this section we stated that there is no coherence to the Atlantic tide gauges for PC1, which suggests an origin of the signal within the basin. We thus conjecture that a baroclinic, density‐related effect inside the North Sea is responsible for PC1 and attempt an explanation in terms of known relationships between tidal currents and turbulent energy losses in varying stratification conditions. This attribution primarily arises from considerations at seasonal time scales. Using hydrographic casts and baroclinic model simulations, Müller et al. (2014) linked M_2_ elevation changes of 1–5 cm in the southern North Sea to the see‐sawing of continental shelf stratification between statically stable summer and well‐mixed winter conditions. Strong buoyancy gradients in mid‐depths (20–30 m) of shallow waters arise during summer months (see e.g., van Haren et al., 1999) and stabilize the water column against energy losses to vertical mixing. The associated increase in barotropic tidal transport and surface elevations was found to be most pronounced in very shallow areas and for cyclonic rotation of strong tidal currents (Müller, 2012) – conditions that are all present in the North Sea.

**Figure 9:**
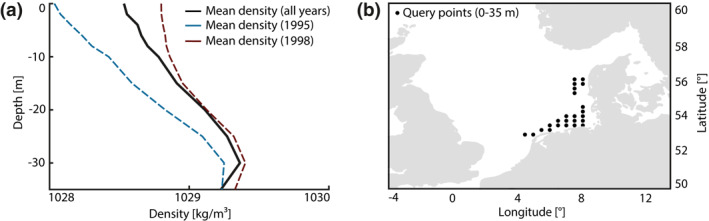
(a) Vertical profiles of potential density as averaged over all query points in (b) at depths from 0 to 35 m for the years 1958–2013 (black), the year 1995 (blue) and the years 1998 (red). The two selected years feature the greatest deviation from the mean density profile.

To relate at least parts of the PC1 content to this process, we have analyzed the temporal evolution of the North Sea’s density structure based on gridded temperature and salinity profiles from the KLIWAS data set (Bersch et al., 2016). These data are provided as annual values through to 2013 at comparatively high spatial resolution (0.25 × 0.5° latitude‐longitude boxes, 2–5 m depth intervals). For consistency, the monthly PC1 series was binned to annual values (1958–2013 with respect to the length of the KLIWAS data set) and cleaned from low‐frequency with periods longer than 30 years. Because it is unknown how well KLIWAS represents the smaller, more subtle changes of density across the water column over several decades, we limit our comparison between stratification and PC1 to variability on interannual time scales. To suppress noise in the climatology, vertical density profiles from a particular set of grid points around the German Bight were averaged to a mean water column structure per year (Figure 9). These query points, indicated by black dots in Figure 9b, lie within 2° of 54.5°N/6.0°E and have an exact depth of 35 m in the KLIWAS data set. The sampled area is shallow, hosts strong tidal currents, and is not permanently mixed, thus favoring a potential effect of stratification on tides. The corresponding time‐averaged density profile (Figure 9a) indicates a pycnocline at 20–25 m, conforming in principle to modeling results (e.g., Guihou et al., 2018; van Leeuwen et al., 2015). While this agreement is reassuring, we also note that our crude spatial averaging ingests profiles in various states of stratification (i.e. homogeneous, seasonally or intermittently stratified conditions, see Van Leeuwen et al., 2015). Given the tendency for in situ measurements being taken in summer, the KLIWAS data set may, however, mainly represent the seasonally stratified case.

**Figure 10:**
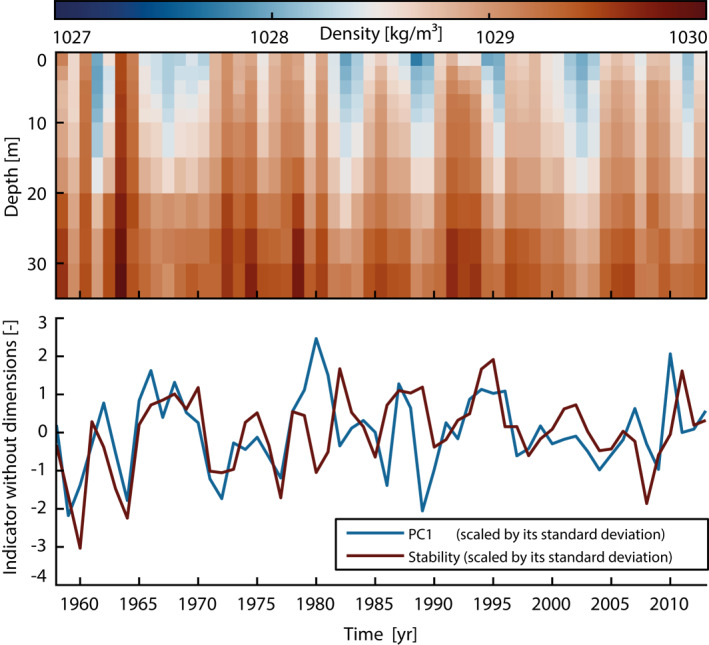
(a) Spatially averaged density profiles (0–35 m) from the query area in Figure 9b spanning the period 1958–2013. (b) Comparison between PC1 changes and the stability index (see main text), where both time series were scaled by their standard deviation and adjusted for long‐term trends.

Some interannual variability in density gradients is already evident from Figure 9a, where we plot individual profiles for the years 1995 and 1998, which differ markedly, by almost 1 kg/m^3^, near the surface. An extension to the full depth‐time sequence (1958–2013, upper 35 m, see Figure 10a) suggests that fluctuations of this magnitude are common but the density perturbations are often mixed throughout the water column, making it difficult to align stratification changes in particular years to highs or lows in the PC1 series. We therefore define an approximate stability index as top‐to‐bottom stratification (cf. Eq. 2.9 of Knauss & Garfield, 2017)

(3) $$\mathrm{Stability}=\frac{\rho_{\mathrm{top}}-\rho_{\mathrm{bed}}}{\delta H}$$

where $\rho_{\mathrm{top}}$ is the averaged density over depths 0, 2 and 4 m, $\rho_{\mathrm{bed}}$ is a mean density across 25, 30, and 35 m, and δH=28m. The adopted metric is akin to the potential energy anomaly advocated by Simpson (1981) and expresses the shape of the density profile through its first derivative. From Figure 10b, we see that the stability index exhibits some noticeably similarity with interannual tidal range changes in PC1. It closely follows the PC1 curve until 1979, echoes the broad peaks around the years 1987 and 1995, and features multiple reversals in sign from 2007 onward. Alongside this qualitative agreement, the observed changes in density gradients amount to about 0.3 kg/m³ per 10 m of depth and thus correspond to the order of magnitude that maintains the seasonal cycle of M_2_ in this region (Müller et al., 2014). Therefore, all indications are that changes to the intensity of summer stratification and/or the time spent in a stratified (or mixed) regime over the course of a year cause the variance in tidal range represented by PC1. When PC1 is multiplied by the corresponding EOF coefficients, we find that for a 1−σ variation in the stability index the tidal range at tide gauges in the Southern German Bight changes by 2.4–2.7 cm, depending on location.

A breakdown of our results into different modes of stratification variability is tempting but beyond the scope of our study as it would call for consideration of several factors, including freshwater buoyancy input, variable local wind stirring, and the inflow of Atlantic water masses through the northern and southern boundaries (Mathis et al., 2015). Nevertheless, we have analyzed long‐term hydrographic data of the North Atlantic and detected high negative correlations (−0.8) between PC1 and temperature of the upper ocean off the Scottish (down to about 300 m) and Norwegian coasts (150 m). The anti‐correlation is most pronounced in individual years prior to the 1990s and still persists on decadal time scales. This preliminary finding suggests that a wider North Atlantic scope must be adopted to unravel the origin of the North Sea tidal range changes, including the observed trends.

## 5 Summary and Conclusion

We have shown that the tidal range in the southwest and the southeast of the North Sea is characterized by a dipole‐like pattern between 1958 and 2014, indicating that different forcing mechanisms of shelf‐wide or larger spatial character may have been present. To separate these processes, and treat both trends and short‐term variability in a unified framework, a PCA‐based method was applied to 70 monthly time series of tidal range throughout the North Sea between 1958 and 2014. Data gaps were filled by the statistical method of Ordinary Kriging. A special property of the Kriging procedure is the conservative nature of its estimates at query points, resulting in under‐rather than over‐estimation of the general system behavior with regard to trends and PCs. We were able to detect two large‐scale signals and explain about 69% of the overall variability in the study area. We attribute the remaining variability of 31% to local effects, which vary widely; they may be absent or could well cause over 50% of variability at an individual tide gauge. In the overall variance, the maximum contribution of a single local effect is at 4%, the average is below 0.4%.

The second PC represents a large‐scale barotropic signal and accounts for the negative trends in the United Kingdom area (up to −2.3 mm/yr). This mode of variability has a North Atlantic extent, as shown by supplementary analysis of tide gauges in Canada, Reykjavik, and the European Atlantic coast. Correlations across the basin are high (0.5–0.7) and are caused by common oscillations on time scales between 6 and 24 months. By detecting the same barotropic signal in the shallow‐water model of Arns et al. (2015a, 2015b), and eliminating suspects that are not part of the model input or physics, we conclude that only sea level rise and meteorological forcing remain as possible causes. However, no linear correlations with these parameters were found, implying that non‐linear interactions must be present. A further indication for the presence of shallow water effects is the severe weakening of the signal as the tidal wave advances from the relative deep water at the United Kingdom into the shallow water areas at the southern and the eastern boundaries of the North Sea.

The absence of PC1 in the barotropic model and its confinement to the southern North Sea coast has prompted us to hypothesize that local stratification changes exert a strong influence on the tidal range in shallow water at various time scales. By analogy to the known seasonal tidal cycle in the area (Müller et al., 2014), we argue that a stronger pycnocline, possibly lasting over longer periods, stabilizes the water column against turbulent dissipation and allows for higher tidal elevations at the coast. The qualitative and quantitative agreement between inter‐annual PC1 changes and an empirically derived stability index is certainly tentative, yet it provides an attractive first‐order target for more systematic data analysis and numerical modeling. Further insight into the nature of large German Bight tidal range changes—particularly the underlying trends—could be furnished by a regional general circulation model with realistic background flow and open boundaries to the North Atlantic.

## Data Availability

Data from GESLA (Global Extreme Sea Level Analysis, GESLA Version 2P. Woodworth et al. 2017), Open Earth (Deltares, http://opendap.deltares.nl/thredds/catalog/opendap/rijkswaterstaat/waterbase/27_Waterhoogte_in_cm_t.o.v._normaal_amsterdams_peil_in_oppervlaktewater/nc/catalog.html), and the responsible German authorities (Wasser‐ und Schifffahrtsverwaltung des Bundes via the portals of the associated Central Data Management, ZDM, https://www.portalnsk.de) were used.
